# Ischemic Stroke Secondary to Dynamic Vertebral Artery Stenosis: Case Report and Review of the Literature

**DOI:** 10.7759/cureus.20167

**Published:** 2021-12-04

**Authors:** Mohammed K Bukhari, Saeed A Alghamdi

**Affiliations:** 1 Neurology, College of Medicine, King Saud Bin Abdulaziz University for Health Sciences, Jeddah, SAU; 2 Research, King Abdullah International Medical Research Center, Jeddah, SAU; 3 Neurology, Division of Neurology, Department of Medicine, King Abdulaziz Medical City, National Guard Health Affairs, Jeddah, SAU

**Keywords:** ischemic stroke, dynamic vertebral artery stenosis, vertebral artery occlusion, bow hunter syndrome, vertebral artery compression, stroke

## Abstract

Ischemic stroke secondary to dynamic vertebral artery stenosis or occlusion, also known as “bow hunter's syndrome,” is a rare stroke mechanism. We report a case of a 24-year-old man with multiple hereditary exostosis (MHE) diagnosed at childhood. His first presentation to a neurologist was due to neck pain and clinical syndrome suggestive of ischemia in the vertebrobasilar territory. A therapeutic occlusion was done successfully without complication. The patient was discharged two days later on aspirin alone. In follow up one year later he continued to be symptom free. Moreover, this stroke mechanism has been reported extensively in the literature in isolation or secondary to many underlying diseases. In total, there are 168 cases reported in the published English literature, in either case reports or small series. In this review, we found that by far, vertebral artery occlusion at the atlanto-axial (C1-2) level dominated most reported cases. The most frequent presentation that led to further investigation was syncope or pre-syncope provoked by head rotation to one side. To our knowledge, there is no previous report of any stroke syndrome related to MHE before our case. In this paper, we report the first case secondary to MHE and review the literature up to date since the first reported case in 1952.

## Introduction

Strokes in the vertebrobasilar territory are commonly due to diseases affecting the vessels, such as atherosclerosis, penetrating small-vessel disease, or arterial dissection [1]. Conversely, non-traumatic dynamic rotational occlusion of the vertebral arteries causing an ischemic stroke or recurrent transient ischemic attack (TIA) is very rare. In 1978, the term bow hunter’s stroke was introduced to describe this stroke mechanism [2]. Some of the common symptoms that occur with this syndrome with head rotation are dizziness, nystagmus, and syncope [3]. Since there are no guidelines for the diagnosis of this syndrome, clinicians use different imaging modalities such as cerebral angiogram, magnetic resonance angiography, ultrasonography, or computed tomography angiography [3]. Moreover, the treatment for this syndrome is conservative management that includes neck immobilization or invasive treatment like surgical decompression.

We will present a case of a young man with multiple hereditary exostoses (MHE) diagnosed during childhood, presenting with a minor ischemic stroke followed by TIA related to head rotation. His vascular imaging revealed a dynamic severe narrowing of the left vertebral artery on head-turning to the right, with reproducibility of his symptoms. He was treated by endovascular occlusion of the culprit vessel.

## Case presentation

A 24-year-old man with MHE was diagnosed in childhood. His syndrome is the result of a *de novo* gene mutation and has been associated with multiple exostoses (also known as osteochondroma) mainly involving his extremities. He underwent several surgeries in the past to remove these bony lesions that have caused minor disability and moderate pain. He had no established stroke risk factors and has been otherwise healthy.

**Figure 1:**
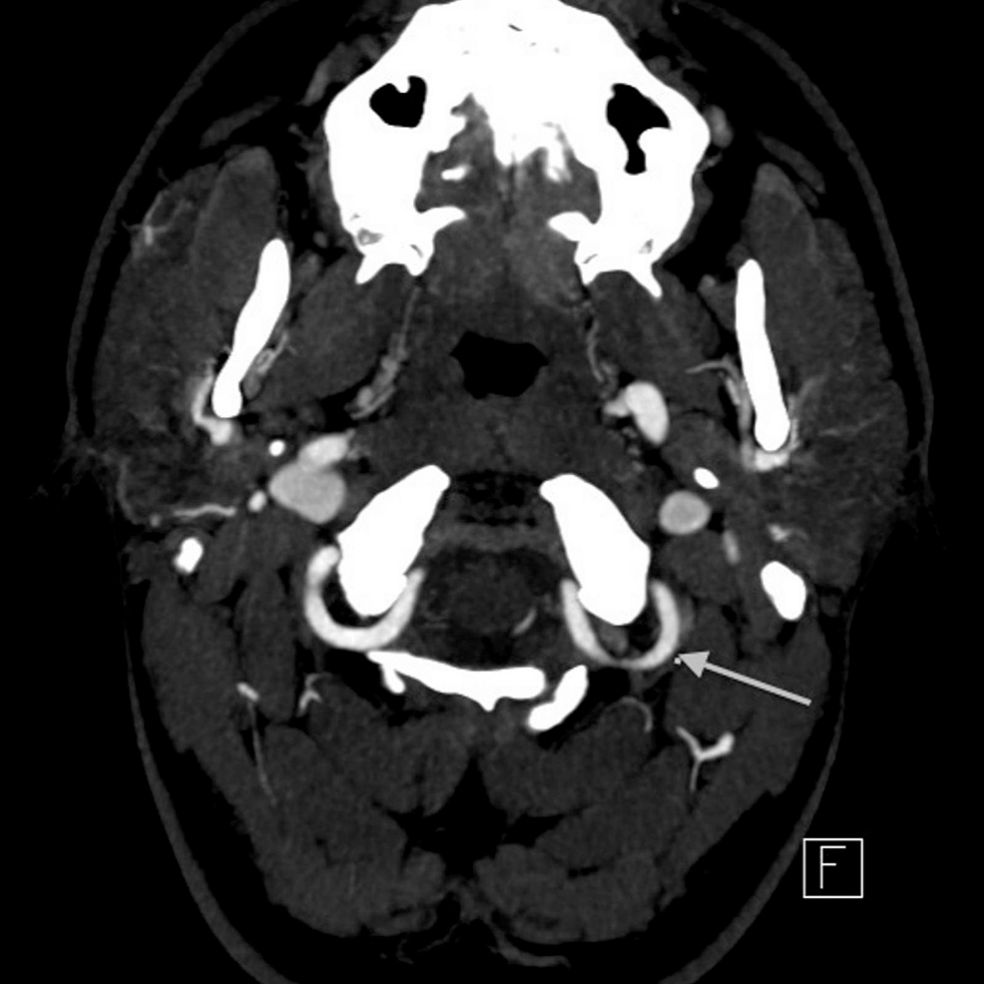
CT angiography (CTA) axial view shows severe narrowing of the left vertebral artery at C2 level with possible dissection (arrow).

**Figure 2:**
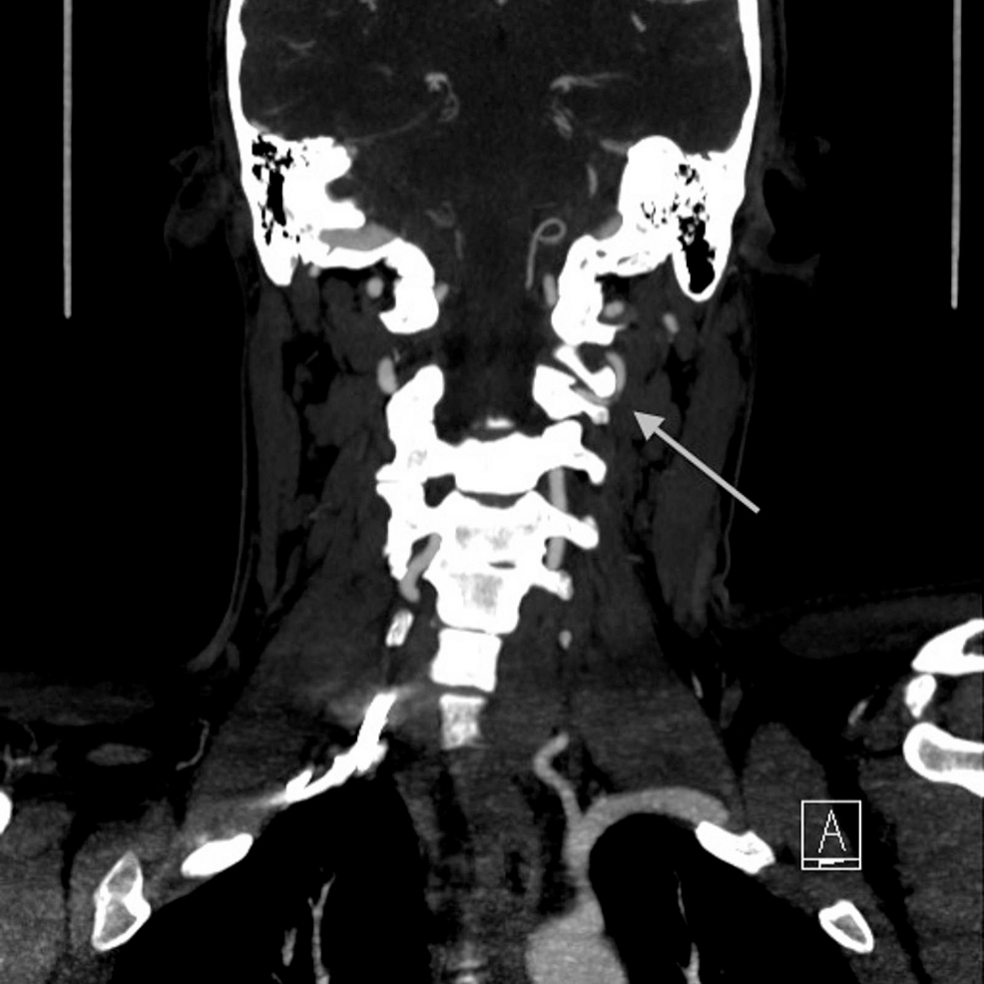
CT angiography (CTA) coronal view, shows severe narrowing of the left vertebral artery at C2 level with possible dissection (arrow).

**Figure 3:**
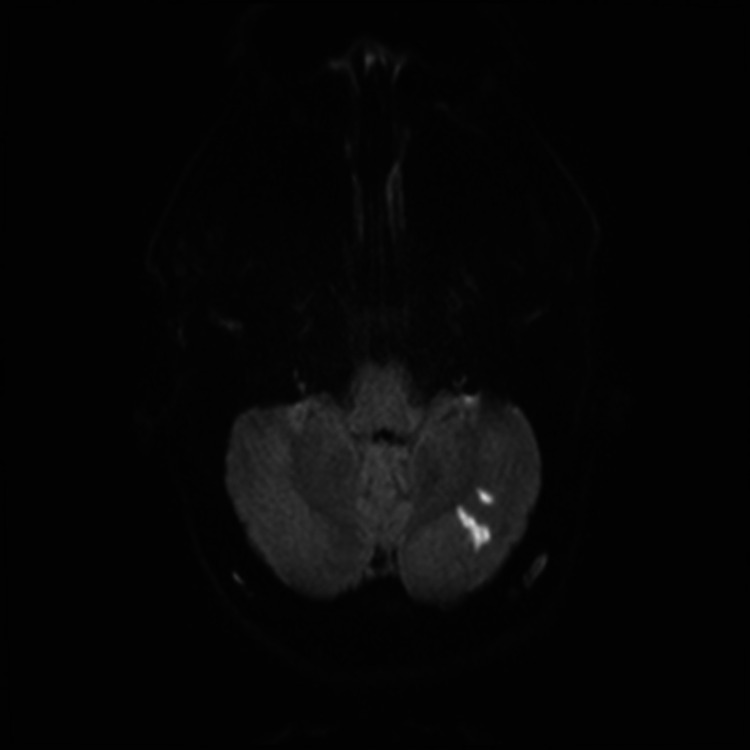
MRI of the brain with diffusion-weighted images (DWI) shows small infarctions at the left cerebellum.

**Figure 4:**
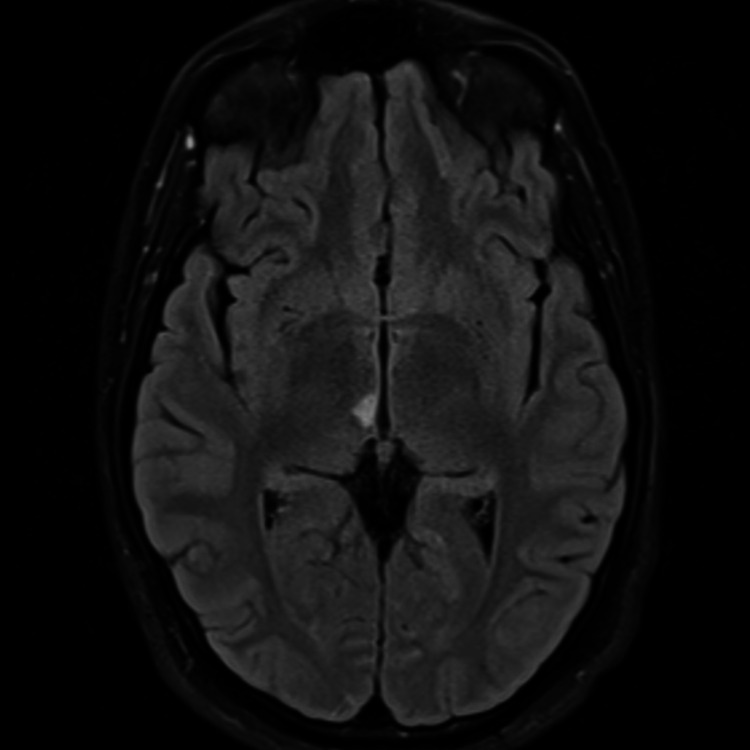
MRI of the brain with T2 weighted image shows small infarctions at the right thalamus.

**Figure 5:**
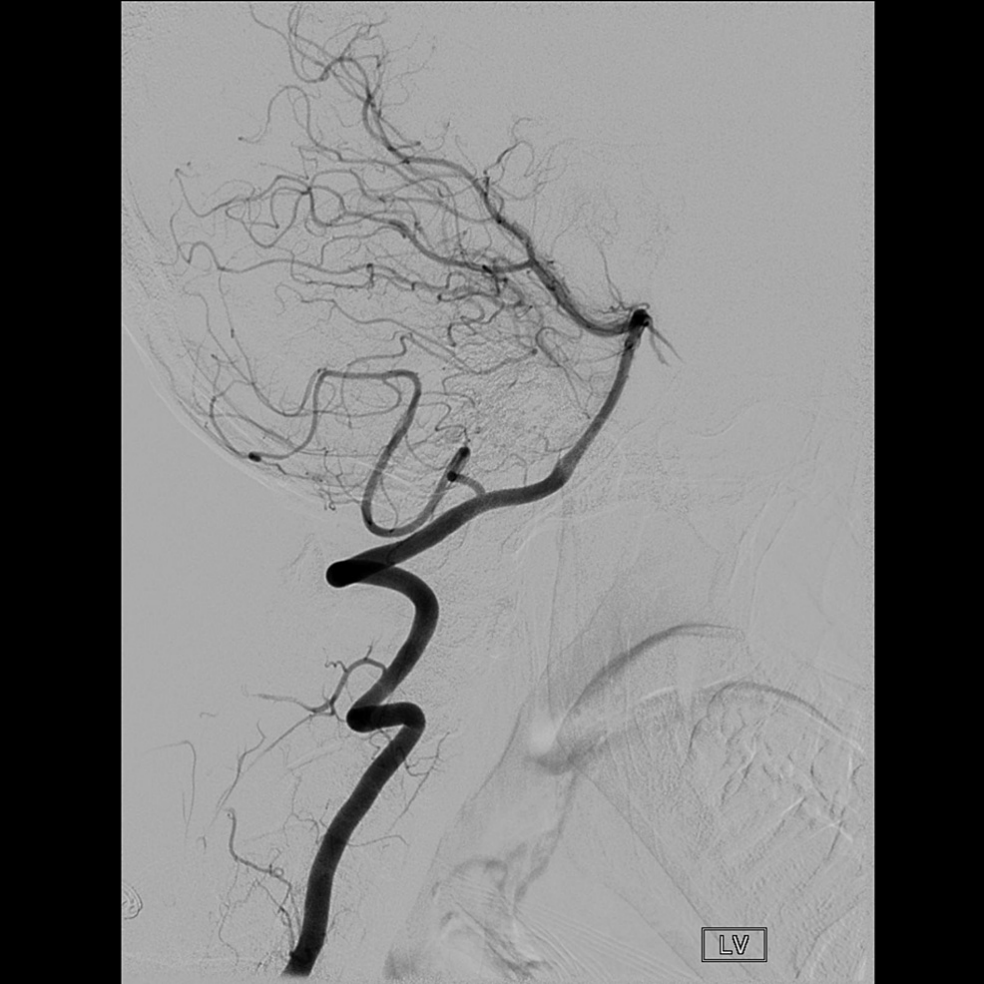
Cerebral angiogram with head in the neutral position.

**Figure 6:**
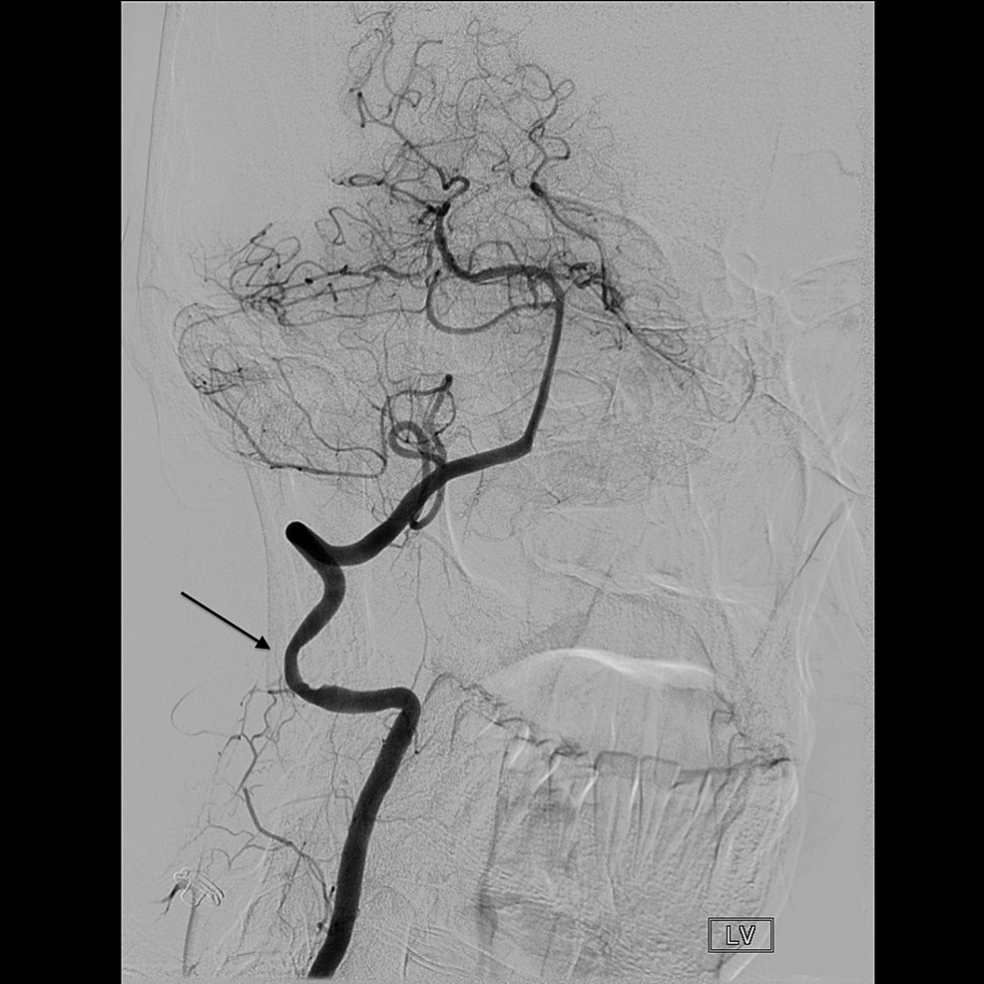
Cerebral angiogram with the head rotated to the right shows the dynamic stenosis of the left vertebral artery on head-turning (arrow).

His first presentation to a neurologist was two weeks prior to admission. This was when he presented with neck pain and clinical syndrome suggestive of ischemia in the vertebrobasilar territory. He denied any history of trauma or neck manipulation. His main neurological finding at that time was persistent limb ataxia on the left side. His cranial computed tomography (CT) and CT angiography (CTA) reported no parenchymal or vascular abnormality. However, it showed multiple exostoses growing of his vertebrae at C1 - C2 level with narrowing of the vertebral canal. MRI with diffusion-weighted images (DWI) showed restricted diffusion in the left cerebellum and right thalamus. He was discharged on aspirin and scheduled for follow-up. One week after his discharge, he presented to our institution complaining of recurrent isolated spells of loss of vision on the left visual field. His neurological examination showed left superior homonymous quadrantanopsia that lasted only a few hours. On the contrary, the left arm ataxia persisted. Repeat CT of the head showed no evolution of the previous stroke, and CTA demonstrated a left vertebral artery dissection at C2 level. The vertebral exostoses were impinging on the vessel and causing significant narrowing (Figures 1, 2). His neck was immobilized, clopidogrel was added to aspirin, and later he was anticoagulated with fractionated heparin as he continued to experience TIAs manifested by recurrent left quadrantanopsia, left facial numbness, and worsening of limb ataxia on the left while being on dual antiplatelets. A new MRI of the head showed no new infarcts and confirmed the presence of the previously reported restricted diffusion in the left cerebellum and right thalamus (Figures 3, 4). Surgical decompression on his cervical spine was deferred because it was considered a high-risk operation. Thus, the decision was made to perform a cerebral angiogram for consideration of left vertebral artery sacrifice/occlusion. The angiogram confirmed the dynamic nature of the left vertebral artery narrowing with severe stenosis on head-turning to the right (Figures 5, 6). A therapeutic occlusion by a detachable balloon with prior balloon test occlusion was done successfully without any complication. The patient was discharged two days later on aspirin alone. In follow up one year later he continued to be symptom free.

## Discussion

Vertebral artery dissection is a common mechanism for vertebrobasilar strokes especially in the young population [4]. Other rare mechanisms have been described and one of them is the dynamic occlusion of the vertebral artery on head rotation. In a study of 1108 patients undergoing cerebral angiogram for different indications, rotational occlusion of either vertebral artery happened in 5% of the patients. Not all of them were symptomatic and the most predictive symptoms for positive angiogram were fainting and dimming of vision [5]. Husni et al. found that in 23 symptomatic patients with rotational occlusion of either vertebral artery, the other one would be either hypoplastic (22 patients) or critically narrowed at its origin (one patient) [6].

Probably the first described case was the one by Ford in 1952. He described a patient with syncope, vertigo, and disturbed vision provoked by voluntary head rotation. The proposed mechanism was intermittent obstruction of the vertebral artery due to a defect in the odontoid process and excessive mobility of the second cervical vertebra [7]. In 1978 a paper describing vertebrobasilar stroke caused by a similar mechanism in a man while practicing archery introduced the term “bow hunter’s stroke,” which was later adopted in most similar reports in the literature [2]. Many case reports or small series have been published since then, describing patients with different vertebrobasilar stroke syndromes sharing the same mechanism related to head rotational movement. We will list and summarize the findings of those cases published in English at Medline since the case of Sorensen [2] in 1978 (Table 1). In total, there are 168 cases reported in the English literature, in either case reports or small series. In this review, we chose to include only the cases that documented dynamic and symptomatic occlusion of the vertebral artery by cerebral angiogram. Only one case that did not respect this criteria and CTA was the only study performed is included [8]. Although some papers proposed a definition that would only include cases with vertebral artery occlusion at the atlanto-axial level, we thought differently as some other reviewers did and included cases where the vessel was involved at lower cervical or even higher (cranio-cervical junction) levels. 

In this review, we found that by far, vertebral artery occlusion at the atlanto-axial (C1-2) level dominated most reported cases 100 out of 168 (59.52%). The remaining reports describe cases where the vessel occlusion happened at the lower cervical spine level, except two reports that described occlusion due to obstruction at cranio-cervical junction [9,10]. The most frequent presentation that led to further investigation was syncope or pre-syncope provoked by head rotation to one side. Conservative management with antiplatelet or anticoagulant therapy and sometimes with neck immobilization was the option in 23.21%. Only 3.6% of them failed this approach and required some intervention with either fusion or decompressive surgery. In those where the outcome of treatment was reported during follow-up 144 cases out of 168 (85.7%), the surgical intervention by either fusion or decompressive surgery was favorable compared to conservative therapy. Moreover, recurrent symptoms occur in 3%, and stroke happening in 2.4%. We should not draw firm conclusions from this comparison given that most of the literature on this subject is coming from the surgical field and the potential for publication bias is high. Endovascular interventions were only reported in nine cases. The cases treated with endovascular stent are five [11-15]. Moreover, there are four cases treated with coil embolization [16-19].

Many different etiologies were reported causing the external compression of the vertebral artery. Instability or subluxation of the cervical uncovertebral joint at different levels due to degenerative spine disease, rheumatoid arthritis, or trauma was the most common [8,20-25]. Traumatic fracture of the atlas was reported in one case [26]. Some of the other etiologies include longus colli muscle hypertrophy [27], disc herniation [28], occipital bone osseous anomaly [9,10], thick fibrous band [29], cervical vertebra osseous anomalies [30,31], tortuosity in the V1 segment [11], osteophyte formation [32-37], schwannoma [38], congenital bilateral C2 transverse foramina stenosis [39], thyroid cartilage compression [40], facet hypertrophy at C4-5 and associated spondylolisthesis [41], and congenital C2-C3 fusion [42].

Hereditary multiple exostoses (HME) is a genetic bone disease characterized by the development of benign bone tumors and exostoses [osteochondromas] growing off the metaphysis of long bones. It is caused by a mutation in the EXT1 or EXT2 genes, which are both tumor suppressor genes. Most cases are inherited in autosomal dominant trait, and sporadic cases are less often [43]. The most common level for spinal involvement in HME is at C2 level. Neurological complications of this disease are all related to tissue compression by the enlarging exostoses. Nerves, roots, and spinal cord compression have been reported [44]. We will list and summarize the findings of all published cases in English at Medline since the case of Sorensen in 1978 (Table 1). Therefore, to our knowledge, there is no previous report of any stroke syndrome related to HME before our case.

**Table 1:** Summary of all cases of bow hunter’s syndrome reported in the English literature since the first case described by Sorensen in 1978. RV= Right vertebral, LV= Left vertebral, BV= Bilateral vertebral, VA= Vertebral artery, C= Cervical vertebrae, w= week, m= Months, yrs= Years, CT= computerized tomography, 3D CTA= Three-dimensional CT angiography,  TCD= Transcranial Doppler ultrasound, MRI= Magnetic Resonance Imaging, MRA= magnetic resonance angiography, N/A= Not reported in the paper, CVJ= Cranio-vertebral junction, LIC= Left Internal Carotid artery. $ Reported immediate outcome in all cases and further follow up in few of them. * Only 12 out of 21 in the report were typical cases.

Author	Year	No. of cases	Sex	Age	Presentation	Side	Level	Imaging	Treatment	Follow up	Prognosis
Sorensen [2]	1978	1	M	39	Lateral Medullary syndrome	RV	C1-C2	Cerebral angiogram	Conservative	2 w	Good
Kojima et al [27]	1985	1	M	64	Rotational Syncope	RV	C6-C7	Cerebral angiogram	Surgical decompression	1.5 yrs	Good
Yang et al [45]	1985	2	2M	Mean 58	Episodic blindness & Presyncope	2 LV	All C1-C2	All Cerebral angiogram	1st C1-2 fusion 2nd Conservative	Both 6 m	Good
Shimizu et al [46]	1988	1	M	37	Bilateral Cerebellar strokes	LV	C1-C2	Cerebral angiogram	Surgical decompression	2 yrs	Good
Hanakita et al [47]	1988	3	3F	Mean 58	Rotational Vertigo. Hemiparesis Drop attacks	2 RV 1 LV	All C1-C2	All Cerebral angiogram	All Surgical decompression	2 yrs	Good
Fox et al [48]	1995	1	F	53	Tinnitus, syncope	LV	C1-C2	Cerebral angiogram	Surgical decompression	6 m	Good
Morimoto et al [49]	1996	1	M	70	Rotational vertigo and Syncope	LV	C1-C2	3D CTA & Cerebral angiogram	C1-2 fixation	N/A	Good
Matsuyama et al [50].	1997	17	7F 10M	Mean 61	7 vertigo 4 dizziness 5 syncope 1 numbness	5 RV 12 LV	All C1-C2	All Cerebral angiogram	8 fusion at C1-2 9 Surgical decompression	Variable$	In the decompression arm 2 had recurrent symptoms, and 1 had cerebellar infarction.
Kawaguchi et al [22]	1997	1	M	56	Rotational blindness	RV	C4-C5	3D CTA & Cerebral angiogram	Surgical decompression	N/A	Good
Matsuyama et al [51]	1997	1	M	71	Vertigo Syncope	LV	C1-C2	3D CTA & Cerebral angiogram	C1-2 fusion	N/A	Good
Kimura et al [24]	1999	1	N/A	N/A	Vertigo Paresthesia	BV	R C5-C6 L C1-2	Cerebral angiogram	C5-6 fusion	N/A	Good
Shimizu S [26]	1999	1	M	53	Vertigo and fainting.	LV	Atlas	Cervical angiogram, 3D CTA, and CT.	Surgical Decompression.	N/A	Good
Sakai et al [52]	1999	1	M	39	Rotational Syncope	RV	C1-C2	3D CTA & Cerebral angiogram	Conservative	N/A	N/A
Seki et al [53]	2001	1	M	47	Rotational Syncope	LV	C1-C2	Cerebral angiogram	Surgical decompression	6 m	Good
Vates et al [28]	2002	1	M	56	Rotational Syncope	LV	C4-C5	Cerebral angiogram & TCD	Discectomy	6 w	Good
Horowitz et al [54]	2002	1	M	55	Rotational Syncope	RV	C1-C2	Cerebral angiogram & TCD	Conservative	N/A	N/A
Tominaga et al [9]	2002	1	M	34	Recurrent strokes	LV	CVJ	Cerebral angiogram	Surgical decompression	N/A	Good
Kamouchi M [55]	2003	2	F M	54 77	Rotational Syncope.	2 LV	V2 N/A	Cerebral angiogram and doppler ultrasonography.	C1-C2 posterior fixation with decompression.	N/A	N/A
Netuka et al [56]	2005	1	M	54	Rotational Syncope	LV	C1-C2	Cerebral angiogram	Surgical decompression	2 yrs	Good
Iguchi et al [57]	2006	1	M	45	Rotational syncope	RV	C2-C3	Cerebral angiogram & TCD	Conservative	N/A	Good
Velat et al [35]	2006	1	M	58	Rotational syncope	LV	C5-C6	Cerebral angiogram	Surgical decompression	4 w	Good
Bulsara et al [32]	2006	1	M	55	Rotational syncope	RV	C5-C6	3D CTA & Cerebral angiogram	Discectomy and foraminal decompression	6 w	Good
Whitmore et al [37]	2007	1	M	57	Rotational syncope	LV	C1-C2	Cerebral angiogram & TCD	Surgical decompression	6 m	Good
Tsutsumi et al [30]	2008	1	M	59	Rotational syncope	BV	R C6 L C6	Cerebral angiogram & TCD	C5-7 fusion	N/A	Good
Kim et al [58]	2008	1	M	60	Rotational dizziness	RV	C2-C3	3D CTA & Cerebral angiogram	Surgical decompression	1 m	Good
Miele et al [36]	2008	1	M	48	Rotational syncope	LV	C4-C5	Cerebral angiogram	Discectomy and fusion	N/A	Good
Sugiu et al [12]	2009	1	M	56	Rotational syncope	RV	C1-C2	Cerebral angiogram	Stenting stenosis in LV	6 m	Good
Lu et al [10]	2009	1	M	12	R thalamic stroke	RV	CVJ	Cerebral angiogram	Surgical decompression	6 m	Good
Natello et al [13]	2009	1	M	76	Rotational syncope	RV	C4-C6	Cerebral angiogram	Stenting RV Intrinsic stenosis	N/A	Good
Chough et al [33]	2010	1	F	71	Rotational vertigo	LV	C1-C2	Cerebral angiogram	C1-2 fusion	N/A	Good
Saito et al [59]	2010	1	M	7	Recurrent strokes	LV	C1-C2	Cerebral angiogram & TCD Mural Thrombus	C1-2 fusion	10 m	Good
Greiner et al [31]	2010	1	M	15	Recurrent strokes	RV	C1-C2	3D CTA & Cerebral angiogram	Conservative then Surgical decompression	3 m	Good
Saito et al [25]	2010	1	M	26	Recurrent strokes	RV	C1-C2	3D CTA & Cerebral angiogram and TCD	Conservative	1 yrs	Recurrent asymptomatic stroke
Yoshimura et al [23]	2011	1	M	64	Rotational syncope	BV	R C3-C4 L C1-C2	3D CTA & Cerebral angiogram	Conservative then discectomy and fusion at C3-4	3 m	Good
Darkhabani MZ [15]	2011	4	4 M	Mean 69	Rotational vertigo, Syncope, and diplopia.	3 LV 1 RV	N/A	dynamic digital subtraction angiography [DSA].	All stent placement in V2 and 1 had another V1 stent.	Mean 6 m	All good
Sakamoto et al [19]	2011	1	M	16	Recurrent strokes	LV	C1	3D CTA & Cerebral angiogram	Coil embolization of the left VA	10 m	Good
Lee et al [60]	2011	2	1F 1M	Mean 39	Rotational syncope, Ataxia & blurred vision	1 LV 1 BV	1 at C7 1 at C1-C2 & C7	MRA, 3D CTA & Cerebral angiogram	Surgical decompression	4 m N/A	Both good
Shetty [8]	2012	1	F	18	Cerebellar stroke	LV	C1-C2	CTA	Surgical decompression followed by Conservative	N/A	Good
Andereggen L [61]	2012	1	F	66	Rotational vertigo, vomiting, and syncope.	LV	C5-C6	CTA, MRA, and ultrasound.	Surgical decompression.	6 m	Good
Yamaguchi et al [62]	2012	1	M	47	Neck pain	RV	C1-C2	MRA & Cerebral angiogram	Conservative	N/A	N/A
Fujiwara et al [20]	2012	1	M	70	Recurrent strokes	LV	C1-C2	Cerebral angiogram	C1-2 fusion	N/A	Good
Cornelius et al [63]	2012	5	1F 4M	Mean 24	Vertigo, blurred vision, syncope, and 1 infarction	3 LV 2BV	All C1-C2	All Cerebral angiogram	3 Surgical decompression 1 Fusion	1 for 7 m 4 N/A	All Good
Dargon et al [29]	2013	1	M	53	Rotational syncope	BV	R C4-C5 L C1-C2	TCD & Cerebral angiogram	Surgical decompression of RV	6 m	Good
Ding D [64]	2013	1	F	43	Rotational pre-syncope and syncope.	LV	C4-C5	Cerebral angiogram and CTA.	Surgical decompression	N/A	Good
Go G [3]	2013	2	2F	50 42	Rotational vertigo, dizziness, right upper extremity tingling sensations, and syncope.	2 LV	C1 C1-C2	CT angiography and Cerebral angiogram	2 Surgical decompression.	2 N/A	2 Good
Piñol I [65]	2013	1	M	27	Rotational vertigo and dizziness.	RV	C6-C7	MRA and dynamic angiogram.	Cervical arthrodesis.	15 m	Good
Inamasu et al [21]	2013	1	M	22	Cerebellar stroke	RV	C1-C2	CTA & Cerebral angiogram	C1-2 fusion	9 m	Good
Fleming et al [34]	2013	1	M	54	Rotational syncope, vertigo, and tinnitus.	BV	C4-C5	CTA & Cerebral angiogram	Surgical decompression of BV and fusion	3 m	Good
Anene-Maidoh T [18]	2013	1	M	16	Right sided numbness, dysphagia, and right peripheral visual field loss.	RV	C1	CTA. Cerebral angiogram, and MRA.	Conservative then surgical decompression then coil embolization in the RV.	3 m	Good but with some residuals.
Choi et al * [66]	2013	12	5F 7M	Mean 62	Rotational syncope, vertigo, and tinnitus	6 RV 6 LV	All C1-C2	Cerebral Angiogram	10 Conservative 2 Fusion	Mean 45 m	10 good 2 strokes
Zaidi et al [67]	2014	11	5F 6M	58	Rotational syncope, vertigo, and diplopia	3 RV 8 LV	C1-C2 & C5-C7	Cerebral Angiogram	2 Conservative 2 Surgical decompression	Mean 9m	All good
Anaizi AN [68]	2014	1	F	68	disorientation, loss of balance, and occasional loss of consciousness.	LV	C1	MRI, MRA, and Intraoperative fluorescent angiography.	Surgical removal of ventral osteophyte then decompression and mobilization of left vertebral artery	2 m	Good
Sarkar J [69]	2014	1	M	37	Near syncope, tunnel vision, scotomas, and roaring in the ears	RV	C7	Duplex ultrasonography, CTA, formal dynamic angiogram, and MRI.	Conservative.	N/A	Good
Ikeda DS [70]	2014	1	M	44	Continued positional tinnitus, vertigo, nausea, and stroke.	LV	C1	CT, MRI, standard and dynamic diagnostic cerebral arteriography.	Surgical decompression.	3 m	Good
Safain MG [71]	2014	1	F	37	Vertigo, tightness in the right occipital region of her head, headaches	RV	C1-C3	CTA, MRI, and dynamic radiograph.	Surgical fusion.	15 m	Good
Park SH [72]	2014	1	M	35	Recurrent vertigo, visual blurring, nystagmus, and tinnitus.	RV	C1-C2	MRI, CTA , and cervical and cerebellar angiograph, and CT.	Conservative.	N/A	N/A
Takeshima Y [73]	2014	1	F	18	Headache.	BV	C1-C2	3D CTA and MRI.	atlantoaxial posterior fixation with iliac bone graft.	22 m	Good
Buchanan CC [74]	2014	1	M	52	Dizziness, extremity weakness.	LV	C3-C4	Dynamic CTA , and MRI.	Surgical decompression and fusion.	6 m	Good
Yamaguchi S [75]	2014	1	M	45	Rotational vertigo.	LV	C1-C2	MRI, MRA, dynamic angiography, and digital subtraction angiography.	Surgical fusion.	24 m	Good
Schelfaut S [76]	2015	1	M	60	near-syncope, nausea, vertigo and downbeating nystagmus.	RV	C5-C6 & C6-C7	CTA and MRA.	left-side Southwick-Robinson ante- romedial approach, followed by an anterior cervical discectomy and fusion	1 yrs	Good
Yamaoka Y [77]	2015	7	5 M 2 F	Mean 45	Dizziness, vertigo, headache, and 1 truncal ataxia , numbness in the right hand	3 LV 4 RV	3 V3 V3-V4 V4 V4-PICA V1-V2	MRI, MRA, CTA, and ultrasound	N/A	N/A	N/A
Ravindra VM [78]	2015	3	2 F 1 M	Mean 52	Syncope, drowsiness, dysphagia mild right arm ataxia, and loss of consciousness.	1 RV 1 Right PICA	C1 N/A C1	CT, MRI, Cerebral angiography, and Doppler ultrasound.	1- Surgical decompression with laminectomy. 2- right-side temporal craniotomy and resection of the meningioma. 3- a right far-lateral craniotomy.	N/A	N/A
Jost GF [79]	2015	2	1 M 1 F	Mean 51	Syncope, loss of vision, dizziness, and fainting spells.	2 LV	C6-C7 C5-C6	MRI and dynamic angiography,	Surgical decompression and fusion for both.	6 m N/A	Minor symptom. Neck pain and stiffness.
Healy AT [80]	2015	1	M	58	persistent cervicalgia, rotational presyncope, and vertigo.	RV LV	C4-C5 C1-C2	MRI, CT, doppler ultrasound, and dynamic vascular angiography.	Laminectomy and fusion from C2–C6 bilaterally.	1 y	Good
Okawa M [81]	2015	1	F	31	dysarthria and confusion.	RV	C5-C6	MRA, MRI, and 3D CTA.	Surgical decompression.	1 m	Good
Takekawa H [82]	2015	1	F	23	Recurrent ischemic stroke.	BV	C1-C2	MRA, MRI, and echocardiography.	Conservative.	N/A	N/A
Wu R [83]	2015	1	M	40	Dizziness, headache, and vomiting.	RV	C6-C7	CT, MRI, angiography and 3D CTA.	Decompression with conservative therapy.	N/A	Good
Thomas B [16]	2015	1	M	60	Recurrent transient ischemic attacks.	RV	C5	CTA, MRI, dynamic angigram,	Conservative then endovascular coil embolization.	12 m	Good
Nguyen HS [84]	2015	1	M	52	Rotational presyncope and see black spots.	RV	N/A	CTA, cerebral angiogram, and MRI.	Surgical decompression of the vertebral artery.	N/A	Good
Chaudhry NS [85]	2016	2	1 F 1 M	Mean 65	Rotational Syncope, lightheadedness, radiculopathy symptoms in the left hand, and vertigo.	RV LV	C5-C6 C4-C6	CT, CTA, MRI/MRA, and digital subtraction angiography.	Surgical decompression, partial discectomy and resection of the uncovertebral joint at C5–C6 on the right.	6 m 3 m	Both Good
Kageyama H [86]	2016	2	2 M	Mean 17.5	Partial visual field defect and visual disturbance.	BV. RV.	C1-C2 both.	MRI, MRA, Cerebral angiography, and ultrasound.	Posterior fixation both.	N/A	Both Good.
Ariyoshi T [87]	2016	1	M	62	Rotational vertigo and pre-syncope.	RV	C2	Ultrasound, Digital subtraction angiography, and 3D CTA.	Conservative.	N/A	N/A
Brinjikji W [88]	2016	1	M	60	Rotational vertigo, tinnitus, blurred vision, left hemibody numbness, and occasional syncope.	Left internal jugular vein	N/A	MR Venography, CTA, and angiography.	Surgical decompression.	N/A	Good
Felbaum DR [89]	2017	1	M	50	Rotational vertigo, neck pain, and near-syncopal episodes.	BV	C3	MRI, dynamic x-rays, CTA, and Digital subtraction angiography.	Instrumentation from C2 to T2.	1 yrs	Good
Buch VP [90]	2017	1	M	38	Rotational dizziness and presyncope.	RV	C1	CTA, MRA, MRI, digital subtraction angiography.	Surgical decompression.	N/A	Good
Lu T [91]	2017	1	M	71	chronic vertigo, occipital headaches, extremity tremors, and irregular respiration.	BV	C4-C5	Dynamic CT, and X-ray angiography.	Surgical decompression.	1 yrs	Good
Haimoto S [92]	2017	1	M	71	Rotational dizziness and loss of consciousness.	LV	C5-C6	X-ray, cerebral angiography, CTA, and CT.	Surgical decompression and removal of the bony mass.	6 m	Good
Motiei-Langroudi R [11]	2017	1	N/A	61	Rotational lightheadedness and facial numbness.	LV	V1	MRI, CTA, MRA, and digital subtraction angiography.	Conservative then stent.	3.5 m	Good
Berti AF [93]	2017	1	N/A	56	Rotational vertigo, nausea, and diplopia.	RV	C4-C5	CTA, MRI, MRA, and	Endovascular deconstruction.	6 m	Good
Simpkin CT [94]	2017	1	F	59	Rotational dizziness.	RV	V1-V4 C1	MRA.	Facet rhizotomy.	N/A	N/A
Yagi K [95]	2017	1	M	74	Ischemic embolic stroke, vertigo, and visual defect.	LV	C4-C5	CTA.	Surgical decompression and fusion.	N/A	Good
Kitahara H [96]	2017	1	M	83	Dizziness.	LV	N/A	CT, MRI, and ultrasound.	Conservative.	N/A	N/A
Johnson SA [97]	2017	1	M	42	Transient right hemiparesis and right- sided vision loss.	RV	C4-C5	Digital subtraction angiography, CTA, and dynamic imaging.	Conservative then surgical decompression.	8 m	Good
Gordhan A [39]	2017	1	M	41	Rotational dizziness.	BV	C2	CTA and MRI.	Conservative.	N/A	N/A
Bergl PA [98]	2017	1	M	62	Rotational dizziness.	LV	C6	CTA, angiography, and MRA.	Surgical fixation and fusion.	N/A	Good
Iida Y [99]	2018	1	M	65	Dizziness and downbeat nystagmus.	LV	C3-C4	MRI, MRA, and Digital-subtraction angiography, and	Surgical decompression and fusion.	N/A	Good
Albertson AJ [100]	2018	1	F	84	Vertigo and postural instability.	BV	C1	X-ray, CT, CTA, MRI, and MRA.	Discharge.	N/A	N/A
Lukianchikov V [101]	2018	1	F	34	Dizziness and loss of consciousness.	RV	C1	CTA, and CT neuronavigation.	Surgical decompression.	6 m	Good
Schunemann V [102]	2018	1	M	60	Dizziness and loss of consciousness.	RV	C3	CTA, MRI, and dynamic cerebral angiography.	Surgical decompression and fusion.	Several months	Good
Ng S [103]	2018	1	M	70	Dizziness, vertigo, fainting, and syncope.	LV	C3-C4	Dynamic CTA, TCD, and Digital subtraction angiography.	Surgical decompression.	8 m	Good
Jadeja N [104]	2018	1	M	24	Dizziness, diplopia ,and disorientation.	RV	C1-C3	MRA, CTA, MRI, and dynamic X-rays.	Conservative.	3 m	N/A
Kameda T [105]	2018	1	M	56	Rotational presyncope and loss of consciousness.	LV	C1	MRI, CTA,	Surgical inferior rim osteotomy of the C-1 and decompression.	4 y	Good
Cornelius JF [106]	2018	1	M	54	Blurring of vision and syncope.	LV	C6-C7	MRI, MRA, CT, TCD, and CTA.	Surgical decompression.	4 m	Good
Cai DZ [107]	2018	1	M	48	Rotational presyncope.	BV	C3-C4 C2-C3	CTA and MRA.	Cervical discectomy and fusion.	4 m	Good
Karle WE [40]	2018	1	F	54	Nausea, vomiting, vertigo.	RV	Compression by the ipsilateral superior cornu of the thyroid cartilage against the transverse process of C4.	CT, CTA, MRI	Laryngoplasty.	2 m	Good
Kan P [14]	2018	1	M	65	Transient right-sided weakness and loss of consciousness.	LIC	N/A	Dynamic cerebral angiogram.	Stent placement.	1 m	Good
Çevik S [108]	2018	1	F	26	Rotational dizziness.	RV	C1-C2	MRI and 3D CTA.	C1 partial hemilaminectomy then opening of the transverse foramens of atlas and axis with lateral part of the posterior tip of the superior articular process of the atlas.	1 y	Good
Park JH [109]	2019	1	M	55	Recurrent vertigo and syncope.	LV	C4-C5	MRI, CT, dynamic angiography, and CTA.	Surgical decompression and fusion.	N/A	Good
Mori M [38]	2019	1	F	43	Rotational mild left arm pain, dysesthesia, and vertigo.	LV	C6-C7	Doppler ultrasonography, MRI, angiography, 3D CTA, and MRA.	she underwent tumor removal with facetectomy and fusion.	6 m	Good
Hernandez RN [110]	2019	1	F	49	Vague neck pain and severe vertigo, nausea, and near syncope.	LV	C1-C2	MRA and CTA.	Surgical decompression and fusion.	6 m	Good
Cohen N [111]	2019	1	F	2	Transient episode of left-side weakness.	RV	C1-C2	cerebral angiogram and CT.	Conservative.	N/A	Good
Tanaka K [17]	2020	1	M	56	Visual blurriness, dizziness, and nausea.	LV	C2	MRI, CTA, and cerebral digital subtraction angiography.	Endovascular occlusion of the culprit left VA by coil embolization.	9 m	Good
Bando K [112]	2020	1	F	13	Transient visual disturbance, hypoesthesia, and paralysis of the left side of the body.	LV	C1-C2	MRI, MRA, and X-ray.	C1-C2 surgical fusion.	8 m	Good
Qashqari H [42]	2020	1	F	6	Headache and fluctuating right-sided weakness.	BV	C1-C2 C2-C3	MRI/MRA and Dynamic angiogram.	Conservative.	2 y	Stable
Shi C [113]	2020	1	M	19	Dizziness, binocular blackness, and disturbance of consciousness.	RV	C2	MRI and Dynamic CTA.	Conservative.	N/A	Good
Yasuyuki Nomura [114]	2020	1	F	47	Rotational vertigo, nausea, nystagmus, and dullness of the right arm.	RV	N/A	MRI, MRA, and 3D-CT.	Conservative.	N/A	Good
Montano M [41]	2021	1	F	79	Rotational pre-syncope, lightheadedness, a ringing in her ears, and darkening of her vision.	LV	C4-C5	CTA, Dynamic provocative cerebral angiography, and MRI.	Cervical spine decompression at C4-5 with anterior cervical discectomy and fusion, but he is now on conservative treatment.	N/A	N/A

The stroke mechanism in our patient is interesting because either vascular injury in the form of vertebral artery dissection or dynamic stenosis of the vessel on head rotation could explain his symptoms. However, more likely both mechanisms have been responsible for his clinical course, with the initial stroke being related to the dissection and the later TIA’s on head-turning related to the dynamic stenosis of the narrow and compromised vertebral artery.

## Conclusions

Although rare, vertebrobasilar stroke can be caused by dynamic vascular occlusion or stenosis. The hallmark of this presentation is that head turning provokes symptoms. Once suspected, dynamic angiography should be done to confirm the diagnosis. Hereditary multiple exostoses can be associated with different neurological complications and ischemic stroke is one of them, which we believe that our case is the first one to report.
